# Modulation of Caffeine Permeation Kinetics in a Skin-PAMPA Model by Probiotic Lysates and Bile Acids

**DOI:** 10.3390/pharmaceutics18060688

**Published:** 2026-05-31

**Authors:** Maja Đanić, Natalija Dedić, Dragana Zaklan, Slavica Lazarević, Bojan Stanimirov, Momir Mikov, Nebojša Pavlović

**Affiliations:** 1 Department of Pharmacology, Toxicology and Clinical Pharmacology, Faculty of Medicine, University of Novi Sad, 21000 Novi Sad, Serbia; slavica.lazarevic@mf.uns.ac.rs; 2 Department of Pharmacy, Faculty of Medicine, University of Novi Sad, 21000 Novi Sad, Serbia; 202058@mf.uns.ac.rs (N.D.); dragana.zaklan@mf.uns.ac.rs (D.Z.); nebojsa.pavlovic@mf.uns.ac.rs (N.P.); 3 Department of Biochemistry, Faculty of Medicine, University of Novi Sad, 21000 Novi Sad, Serbia; bojan.stanimirov@mf.uns.ac.rs; 4 Centre for Biomedical Research, Faculty of Medicine, University of Banja Luka, 78000 Banja Luka, Republic of Srpska, Bosnia and Herzegovina; mikovmomir@gmail.com

**Keywords:** caffeine, probiotic lysate, bile acids, PAMPA, topical delivery, permeation kinetics

## Abstract

**Background:** Caffeine, although widely used in dermatological and cosmetic products, exhibits limited permeability through the *stratum corneum*, highlighting the need for strategies for optimizing delivery. The aim of this study was in vitro investigation of the effects of probiotic bacterial lysates and submicellar concentrations of bile acids on caffeine permeation, with a particular focus on permeation kinetics. **Methods:** Caffeine permeability was evaluated using the Skin Parallel Artificial Membrane Permeability Assay (Skin-PAMPA). Donor and acceptor concentrations were quantified by HPLC at predefined time points (1, 2, 4, 6, and 12 h), followed by calculation of apparent permeability coefficients, cumulative permeation profiles, and interval permeation rates in systems containing probiotic lysates and submicellar concentrations of cholic acid (CA) or deoxycholic acid (DCA). **Results:** Probiotic lysates significantly reduced caffeine permeability (0.98 ± 0.02 × 10^−6^ vs. 1.57 ± 0.14 × 10^−6^ cm/s in the control group) and modified transport kinetics resulting in lower early-phase interval permeation rates and reduced cumulative permeation. Conversely, bile acids increased the apparent permeability of caffeine, with the highest value observed in the DCA group (2.30 ± 0.08 × 10^−6^ cm/s). **Conclusions:** Overall, probiotic lysates and bile acids modulated caffeine permeation across the Skin-PAMPA membrane primarily by reshaping permeation kinetics rather than simply changing overall permeability. Their combined effects may provide a basis for designing topical formulations with tailored permeation profiles.

## 1. Introduction

Topical drug delivery systems have gained increasing attention as versatile platforms for achieving localized therapeutic effects while minimizing systemic exposure [1,2]. The efficacy of dermatological formulations depends not only on the intrinsic physicochemical properties of the active compound but also on the composition, microstructure, and intermolecular interactions within the formulation matrix, which can influence permeation across the skin barrier [3]. The *stratum corneum*, as the outermost layer of the epidermis, represents the primary diffusion barrier and largely determines the kinetics of transport and permeation of substances through the skin [4]. Therefore, understanding the factors that govern membrane transport kinetics may contribute to the rational optimization of topical delivery strategies, particularly in contexts where modulation of permeation behavior rather than maximal transdermal permeation may be desirable.

Caffeine is a widely used bioactive compound in dermatological and cosmetic preparations due to its antioxidant [5], photoprotective [6], and lipolytic properties, as well as its ability to improve skin microcirculation [7]. It is frequently included in formulations intended to improve skin appearance, reduce edema, provide anti-cellulite effects, stimulate hair growth, and protect against UV-induced damage [8]. Despite its broad use, caffeine does not exhibit ideal characteristics for dermal penetration, as it is relatively hydrophilic, with a logP value of approximately −0.07. In addition, caffeine shows unusual solubility behavior in certain non-aqueous solvents [9] and may form aggregates in aqueous media [10]. Consequently, despite its relatively low molecular weight, dermal permeation of caffeine is primarily limited by its hydrophilic character and is further influenced by formulation-related interactions and interfacial processes at the membrane surface.

Numerous approaches have been investigated to optimize caffeine delivery, either to enhance systemic transdermal permeation or to promote its accumulation within specific skin layers for localized action [11,12,13,14]. While classical chemical enhancers primarily aim to increase drug permeation across the skin barrier, modern vesicular and nanoparticulate systems are increasingly designed to modulate transport kinetics and target specific skin layers or hair follicles rather than simply enhance permeation [15]. These considerations suggest that optimization of topical caffeine delivery may involve not only increasing permeability but also controlling permeation profiles over time.

Among membrane-active compounds, bile acids have attracted considerable attention due to their amphiphilic structure and ability to interact with lipid environments [16]. At submicellar concentrations, bile acids are not expected to form micelles [17] but can still alter membrane organization, interfacial tension, lipid packing, and solute distribution, thereby acting as mild and reversible permeation enhancers [18,19,20].

In recent years, growing attention has also been directed toward probiotic-derived ingredients, particularly bacterial lysates, as biologically active components in pharmaceutical and dermocosmetic formulations [21,22]. Clinical and experimental studies have demonstrated that probiotics may improve skin barrier function, reduce irritation, and enhance overall skin condition [23,24]. However, the use of live microorganisms in topical formulations is associated with challenges related to manufacturing processes, formulation stability, and regulatory requirements, prompting increased interest in postbiotic approaches, including probiotic lysates, which retain biological activity while offering improved formulation compatibility [25,26]. Despite extensive research on probiotic-derived products and their immunomodulatory and barrier-supporting effects [23,27], their influence on small-molecule permeation, particularly on permeation kinetics, remains insufficiently explored.

Skin-PAMPA (Parallel Artificial Membrane Permeability Assay) is an artificial membrane system that provides a simplified skin-mimetic platform for investigating passive diffusion and preliminary assessment of skin permeation behavior [28,29]. The model is widely used as a screening tool for evaluating factors affecting membrane transport and permeability.

Based on these considerations, the present study was designed to investigate the effects of probiotic bacterial lysates and submicellar concentrations of bile acids, both individually and in combination, on caffeine permeation across a Skin-PAMPA membrane, with particular emphasis on time-dependent permeation behavior. The novelty of the present study lies not in the individual components themselves but in their combined evaluation within a single Skin-PAMPA system and assessment of their influence on caffeine permeation kinetics.

## 2. Materials and Methods

### 2.1. Chemicals and Reagents

Commercial probiotic capsules (PROBIOTIC^®^, Hemofarm AD, Vršac, Serbia) were used or the preparation of probiotic bacterial lysates. Each capsule contained 5 × 10^9^ viable lyophilized probiotic bacteria, including *Lactobacillus helveticus*, *Lactobacillus rhamnosus*, and *Bifidobacterium longum*. Caffeine, sodium salts of CA and DCA were purchased from Sigma Chemicals Co., (St. Louis, MO, USA). Phosphate-buffered saline (PBS, 1×, pH 7.4) was obtained from Gibco, Life Technologies (Grand Island, NY, USA). Water and methanol of HPLC grade were purchased from J.T. Baker (Phillipsburg, NJ, USA) and were used for preparation of mobile phase and standard solutions.

### 2.2. Preparation of Working Solutions and Calibration Curve

**Figure 1:**
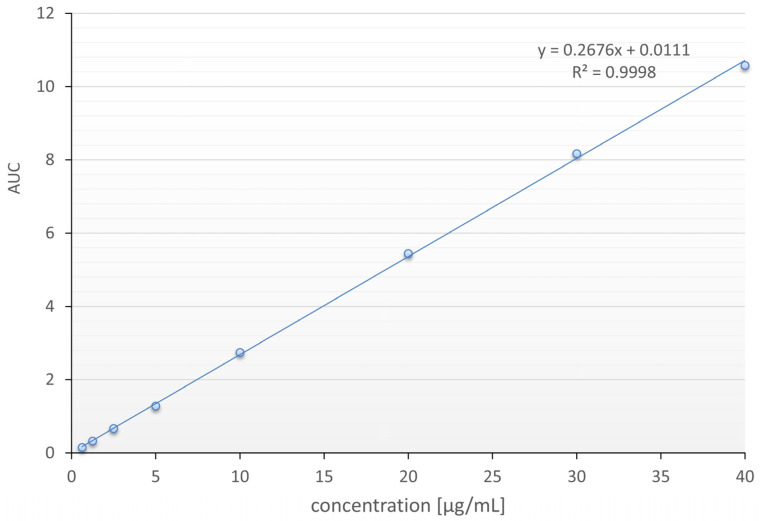
Calibration curve of caffeine standard solutions in the concentration range 0.625–40 µg/mL.

A stock solution of caffeine (1 mg/mL) was prepared by dissolving caffeine in PBS. The stock solution was diluted 2.5-fold with PBS to obtain a working solution with a final concentration of 400 µg/mL. Standard solutions for the calibration curve were prepared by diluting the stock solution with a methanol–water mixture (30:70, *v*/*v*) to obtain concentrations in the range of 0.625–40 µg/mL. Calibration was performed by plotting peak area versus concentration. The calibration curve was linear within the tested range (R^2^ = 0.999) with the regression equation y = 0.2676x + 0.0111. The calibration curve is shown in Figure 1.

### 2.3. Preparation of Probiotic Lysates

Probiotic lysates were prepared according to a previously published method, with slight modifications [30]. The contents of two probiotic capsules were dissolved in 10 mL of PBS. The suspension was subjected to sonication on ice in order to disrupt bacterial cells and release intracellular components. Sonication was performed in three cycles of 30 s, with 60 s intervals between cycles, while samples were kept on ice to prevent overheating. After sonication, the samples were centrifuged at 10,000× *g* for 10 min at 4 °C. The obtained supernatant (lysate) was collected and used for further sample preparation and HPLC analysis. Protein concentration in the bacterial lysates was determined using the Bradford assay with a commercial reagent by measuring absorbance at 595 nm, using bovine serum albumin as the standard. The measured protein concentration in the lysate was 125 µg/mL. Prior to use in the permeation experiments, the lysate was further diluted 1:1 with buffer and used in the donor compartment.

### 2.4. Experimental Groups

To evaluate the effects of probiotic lysates and bile salts on caffeine transport, six experimental groups were prepared:

1. Caff—caffeine solution (200 µg/mL) in phosphate-buffered saline;
2. Caff-C—caffeine solution (200 µg/mL) with sodium cholate (100 µM);
3. Caff-D—caffeine solution (200 µg/mL) with sodium deoxycholate (100 µM);
4. Caff-L—caffeine solution (200 µg/mL) with probiotic lysate;
5. Caff-LC—caffeine solution (200 µg/mL) with probiotic lysate and sodium cholate (100 µM);
6. Caff-LD—caffeine solution (200 µg/mL) with probiotic lysate and sodium deoxycholate (100 µM).

Each experimental condition was assessed in triplicate using three parallel wells within the Skin-PAMPA system.

### 2.5. Skin-PAMPA Permeability Assay

Caffeine permeability was evaluated using the Skin-PAMPA model, which simulates the barrier properties of human skin.

Hydrophobic MultiScreen PVDF 96-well filter plates with a pore size of 0.45 µm (Millipore, Billerica, MA, USA) were used as acceptor plates and as supports for the artificial membrane. The membrane was prepared by impregnating each well of the acceptor plate with 17 µL of a 35% (*v*/*v*) solution of isopropyl myristate and silicone oil (3:7, *v*/*v*) in n-hexane, carefully avoiding contact of the pipette tip with the membrane surface. After solvent evaporation, a lipid artificial membrane was formed.

Each well of the acceptor plate was filled with 300 µL of phosphate-buffered saline (PBS, pH 7.4). The donor plate (MultiScreen Transport Receiver Plate, Millipore, USA) was filled with 300 µL of the test solutions prepared in PBS (pH 7.4). The donor and acceptor plates were assembled to initiate incubation, and the acceptor plate was covered to prevent solvent evaporation.

Samples were incubated at 32 °C under constant orbital shaking (PSU-10i, Boeco, Hamburg, Germany) at 50 rpm, and concentrations were determined at five time points (1, 2, 4, 6, and 12 h). At each time point, the plates were separated, and samples were collected from both donor and acceptor compartments. Caffeine concentrations were determined by HPLC analysis.

Apparent permeability coefficients were calculated using the donor-equilibrium model (P*_app_don_*) and expressed in cm/s according to the following equation:

P*_app_don_* = C × [−ln (1 − [C_a_]/[C_eq_])], (1)

where C = [V_d_ × V_a_]/[(V_d_ + V_a_) × S × t]; (2)

C_a_ denotes concentration in the acceptor compartment;

C_eq_ denotes equilibrium concentration;

V_a_ denotes the volume of the acceptor compartment (mL);

V_d_ denotes the volume of the donor compartment (mL);

t denotes incubation time (s);

S denotes membrane surface area (cm^2^).

The effective membrane surface area was calculated as 0.24 cm^2^, based on the manufacturer’s specification of a filter area of 0.32 cm^2^ and membrane porosity of 75% [31].

For comparison, apparent permeability coefficients were also calculated using the acceptor-only model (P*_app_acc_*) according to the equation:

P*_app_acc_* = (C_a_ × V_a_)/(S × C_0_ × t) (3)

C_a_ denotes concentration in the acceptor compartment;

V_a_ denotes the volume of the acceptor compartment (mL);

S denotes membrane surface area (cm^2^);

C_0_ denotes initial concentration in the donor compartment;

t denotes incubation time (s).

However, the donor-equilibrium model was selected for primary analysis because it incorporates concentration changes occurring in both donor and acceptor compartments during the experiment, thereby providing a more representative description of transport kinetics under the present experimental conditions.

### 2.6. Analysis of Permeation Kinetics

To further characterize permeation kinetics, cumulative permeation and interval permeation rates were calculated. Cumulative permeation was expressed as the percentage of permeated caffeine relative to the initial concentration in the donor compartment (%C_0_). Average interval permeation rates were calculated for two predefined periods corresponding to the early phase (0–6 h) and late phase (6–12 h). The permeation rate in the early interval was calculated as:

R_0–6_ = %_6_h/6 (4)

The permeation rate during the late interval was calculated as:

R_6–12_ = (%_12_h − %_6_h)/6 (5)

Permeation rates were expressed as %C_0_/h and used to evaluate differences in permeation kinetics between experimental groups.

### 2.7. HPLC Analysis

High-performance liquid chromatography (HPLC) equipped with a diode array detector (Vanquish Core HPLC, Thermo Fisher Scientific, Bremen, Germany) was performed according to a previously published and slightly modified method [32]. Separation was achieved on a reversed-phase Zorbax Eclipse Plus C18 column (150 mm × 2.1 mm, 5 µm, Agilent Technologies, Santa Clara, CA, USA) with a Zorbax Extend C18 guard column (12.5 mm × 2.1 mm, 5 µm, Agilent Technologies, USA). The column temperature was maintained at 25 °C and the injection volume was 5 µL. Isocratic elution was carried out using a mobile phase consisting of water and methanol (70:30, *v*/*v*) at a flow rate of 0.35 mL/min, with a total run time of 5 min. Detection was performed at 274 nm, and the retention time of caffeine was 3.14 min.

### 2.8. In Silico Prediction of Skin Sensitization Potential

To provide additional information regarding the safety considerations relevant to topical caffeine exposure, an in silico prediction of skin sensitization potential was performed using publicly available computational models integrated in the SkinSens/PRED-SKIN database. These models are based on alternative non-animal methods accepted by OECD and include prediction of protein reactivity (DPRA/PPRA), keratinocyte activation (KeratinoSens/LuSens), and dendritic cell activation (h-CLAT), which correspond to key events in the adverse outcome pathway (AOP) for skin sensitization. For caffeine, the human skin sensitization probability score and structural alerts related to electrophilic reactivity were assessed [33].

### 2.9. Statistical Analysis

Statistical analysis of the obtained data was performed using IBM SPSS Statistics software (version 21, IBM Corp., Armonk, NY, USA). All experiments were conducted in triplicate, and the results are presented as mean ± standard deviation (SD).

Differences in apparent permeability coefficients between experimental groups were analyzed using one-way analysis of variance (one-way ANOVA), followed by Tukey’s post hoc test for multiple comparisons. This analysis was used to evaluate the effects of probiotic lysates, bile acids, and their combinations on caffeine permeation. Differences in cumulative permeation values between experimental groups at individual sampling time points were analyzed to assess time-dependent permeation behavior. Within each experimental group, interval permeation rates in the early (0–6 h) and late (6–12 h) phases were compared using a paired two-tailed t-test. Differences were considered statistically significant at *p* < 0.05.

## 3. Results

### 3.1. Apparent Permeability Coefficients

**Figure 2:**
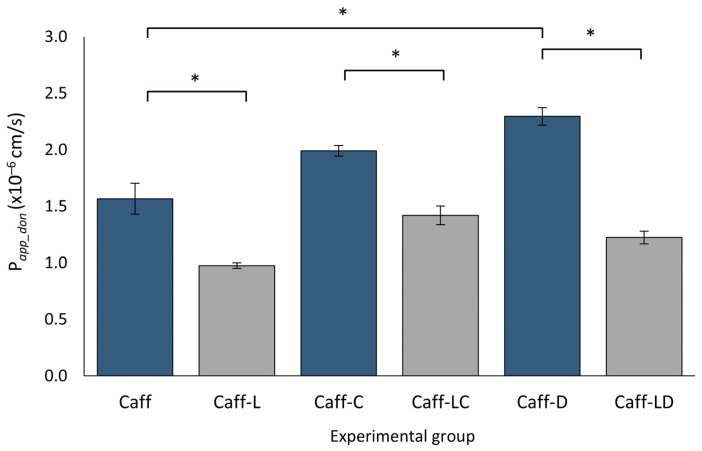
Apparent permeability coefficients (P_app_don_) for all experimental groups (* *p* < 0.05).

Differences in apparent permeability were observed among experimental groups (Figure 2). The highest apparent permeability coefficient (P*_app_don_*) was recorded in the Caff-D group (2.30 ± 0.08 × 10^−6^ cm/s), followed by the Caff-C group (2.00 ± 0.05 × 10^−6^ cm/s), whereas the control group (Caff) showed a value of 1.57 ± 0.14 × 10^−6^ cm/s. In contrast, all lysate-containing groups exhibited reduced permeability, with the lowest value observed in the Caff-L group (0.98 ± 0.02 × 10^−6^ cm/s). Statistically significant differences were identified between Caff and Caff-L (*p* < 0.001), Caff-C and Caff-LC (*p* = 0.002), Caff-D and Caff-LD (*p* < 0.001), and Caff and Caff-D (*p* < 0.001).

**Figure 3:**
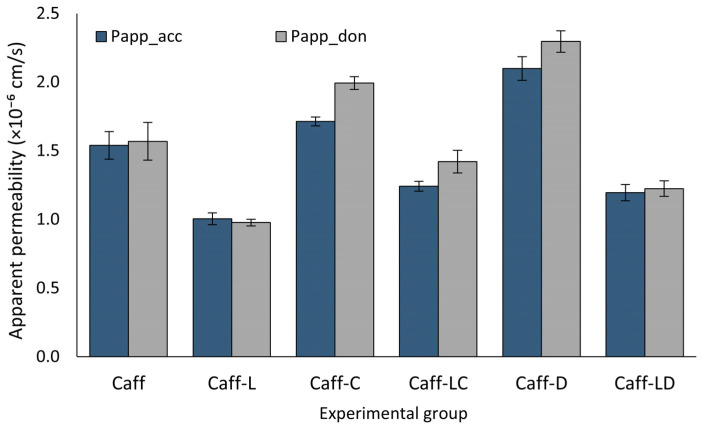
Comparison of apparent permeability coefficients calculated using the acceptor-only model (P*_app_acc_*) and the donor–equilibrium model (P*_app_don_*).

Apparent permeability coefficients calculated using the donor–equilibrium model (P*_app_don_*) were used as the primary permeability parameter. For comparison, permeability coefficients calculated using the acceptor-only model (P*_app_acc_*) were also determined. Although minor differences in absolute values were observed, the overall permeability pattern across experimental groups remained consistent (Figure 3).

### 3.2. Cumulative Permeation Profiles

**Figure 4:**
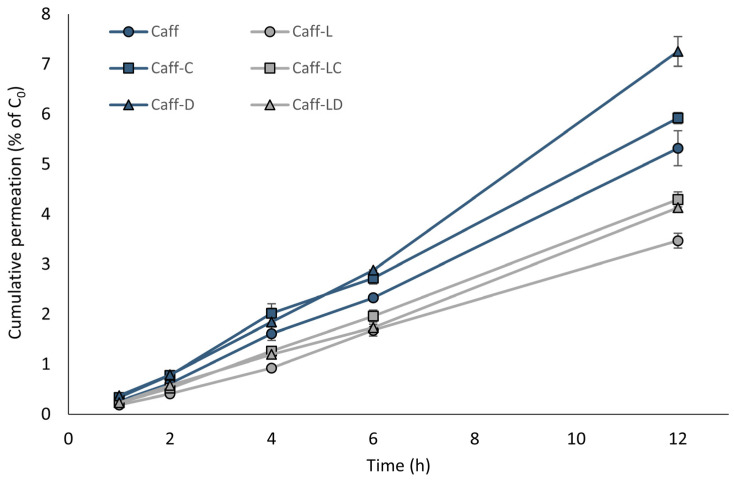
Cumulative permeation profiles of caffeine expressed as percentage of the initial concentration in the donor compartment (C_acc_/C_0_ × 100) for all experimental groups.

Cumulative permeation profiles revealed progressive differences among experimental groups during the incubation period (Figure 4). In all systems, the amount of permeated caffeine increased throughout the 12-h experiment; however, the rate and extent of permeation depended on the composition of the donor phase.

Groups without probiotic lysates (Caff, Caff-C, Caff-D) showed faster permeation rates and higher cumulative fractions of permeated caffeine, reaching up to 7.0% after 12 h. In contrast, lysate-containing groups consistently showed slower permeation, with final permeated fractions ranging from 3.5% to 4.3%. Samples containing bile acids generally exhibited higher cumulative permeation compared with the caffeine-only control.

**Figure 5:**
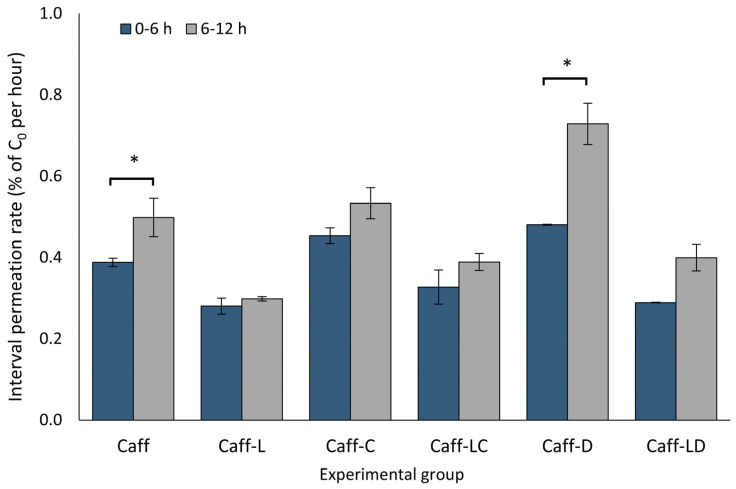
Interval permeation rate of caffeine during the early (0–6 h) and late (6–12 h) phases across experimental groups (* *p* < 0.05).

To further characterize changes in permeation behavior over time, interval permeation rate analysis was performed. As shown in Figure 5, lysate-containing formulations exhibited lower early-phase (0–6 h) interval permeation rates and relatively maintained or increased values during the late phase (6–12 h). In contrast, systems containing submicellar concentrations of bile acids generally showed higher interval permeation rates compared with the caffeine-only control. Comparison of early and late intervals demonstrated statistically significant differences in the Caff (*p* = 0.046) and Caff-D (*p* = 0.038) groups, whereas the remaining groups showed similar trends without reaching statistical significance.

### 3.3. Mass Balance Recovery

Mass balance recovery values for caffeine after incubation ranged between approximately 87% and 108%, indicating acceptable experimental recovery with minimal nonspecific adsorption or degradation. Slightly elevated values observed in some samples were likely associated with analytical variability. Overall, recovery values remained within an acceptable range across experimental groups.

### 3.4. In Silico Prediction of Skin Sensitization

The in silico analysis indicated a high predicted probability of skin sensitization for caffeine, with a predicted human sensitization score of approximately 0.83. The compound was within the applicability domain of the model, implying a reliable prediction. Positive alerts were observed for protein reactivity and activation of keratinocytes and dendritic cells, corresponding to key events in the skin sensitization pathway (Table 1).

## 4. Discussion

The present study demonstrated distinct effects of probiotic lysates and bile acids on caffeine permeation across the Skin-PAMPA membrane, with differences observed not only in overall permeability but also in caffeine permeation kinetics.

A consistent reduction in caffeine permeation was observed in all lysate-containing systems, reflected by lower apparent permeability coefficients and reduced cumulative permeation. In addition, lysates altered the temporal profile of caffeine transport, characterized by lower early-phase (0–6 h) interval permeation rates and a relatively sustained permeation pattern during later stages of the experiment.

One possible explanation may involve changes in the availability of the freely diffusible caffeine fraction within the donor phase. Probiotic lysates contain complex mixtures of proteins, peptides, polysaccharides, and membrane fragments that may participate in reversible intermolecular interactions or alter the physicochemical properties of the surrounding environment [34,35]. In addition, alterations in donor-phase microstructure or local diffusional properties may influence molecular mobility and reduce diffusion toward the membrane interface. Previous studies have shown that the presence of macromolecules in complex matrices can affect molecular mobility and availability for diffusion [36]. Although direct comparison is limited by differences in experimental design, similar phenomena have been reported in structured systems such as microemulsions, where formulation composition and rheological properties influence caffeine permeation kinetics [37].

A potential explanation for the observed lower early-phase permeation rates followed by relatively maintained or increased values during later stages of the experiment may be drawn by analogy with systems involving reversible drug–matrix interactions. Namely, lysate-derived components may transiently interact with a fraction of caffeine, thereby reducing the amount immediately available for diffusion in the donor phase. Consequently, the initial transport rate could be reduced, while subsequent changes in permeation behavior may reflect gradual redistribution of caffeine over time [38]. It may be hypothesized that, as an equilibrium between associated and freely available caffeine becomes progressively established, diffusion through the membrane may proceed at a more sustained rate, resulting in a prolonged permeation profile [39,40]. Such modulation of transport kinetics may be relevant for topical delivery systems where sustained and controlled exposure is desirable rather than maximal penetration across barrier systems.

Although direct studies investigating the effects of probiotic lysates on caffeine permeation are lacking, previous reports suggest that probiotic-derived components may influence skin barrier function and permeability-related processes. For example, lysates of *Lactobacillus rhamnosus* were shown to reduce the penetration of Rhodamine B through reconstructed human epidermis [24]. Similarly, fermented probiotic lysates have been reported to improve skin barrier function and reduce transepidermal water loss, effects that could potentially influence the transport of small molecules across the skin [23].

The increase in caffeine permeability observed in the presence of submicellar concentrations of bile acids may be explained by the amphiphilic properties of these molecules and their ability to interact with membrane-like systems [41,42,43]. Even at concentrations below the critical micellar concentration, bile acids may act as mild and reversible permeation enhancers, increasing membrane permeability without causing complete disruption of barrier integrity [44].

In the present study, systems containing DCA showed higher permeability compared with those containing CA, which is in agreement with previous reports indicating stronger membrane-disrupting effects of more hydrophobic bile acids. The greater lipophilicity of DCA may contribute to stronger interactions with the lipid phase of the membrane, resulting in greater enhancement of diffusion across the barrier [42]. Similar findings with other compounds have been reported in the Skin-PAMPA model, where CA and DCA increased clindamycin permeability, supporting the relevance of bile acids as modulators of passive transport across artificial skin membranes [31].

When probiotic lysates were combined with systems containing bile acids, intermediate permeability values were observed, positioned between lysate-only and bile acid–only systems. This pattern may reflect the simultaneous influence of factors associated with both components on caffeine transport across the membrane. Similar interactions between membrane-active compounds and macromolecular constituents have been described in complex delivery systems, where multiple formulation-related factors may collectively influence the effective transport of the active compound [45]. Accordingly, the resulting permeation profile may depend on the balance between these potentially opposing effects.

While many topical formulation strategies aim to enhance drug permeation across the skin barrier [46], modulation of permeation kinetics may represent an important consideration in the design of dermal formulations, particularly when controlled and sustained exposure to the active compound is desired. This concept is especially relevant for dermatological and cosmetic formulations containing caffeine, where prolonged local activity and limited systemic penetration are often desirable. Although caffeine is generally considered safe for topical use when applied at concentrations up to 3% [8], the in silico predictions obtained in the present study suggest that caffeine may interact with skin proteins and activate cellular pathways associated with skin sensitization. Although the predictive performance of the applied in silico model has been reported as satisfactory, the possibility of false positive results remains a recognized limitation, warranting cautious interpretation of the generated alerts [33]. Nevertheless, isolated clinical observations may provide indirect support for the relevance of such predictions. For example, Tognetti et al. described a case of caffeine-induced urticaria–angioedema after caffeine consumption and suggested that caffeine should be considered a potential urticaria-inducing agent [47].

Overall, these observations further support the concept that maximal skin permeation may not always represent the optimal formulation goal and that modulation of permeation kinetics could be advantageous for achieving controlled and sustained topical delivery.

It should be noted that the Skin-PAMPA model represents a simplified system that primarily reflects passive diffusion and does not fully reproduce the structural and biological complexity of human skin [48]. Nevertheless, the present findings provide preliminary insight into how biologically complex excipients and membrane-active components may influence the transport of small molecules across artificial skin membrane barriers. As direct experimental characterization of caffeine-lysate interactions was beyond the scope of this study, the proposed interpretations remain hypothetical and require further mechanistic investigation. Since the observed permeation behavior may be influenced by both membrane-related factors and the composition of the donor system, the standardization of lysate-containing samples is relevant for interpreting these findings. Total protein content was used as a practical standardization parameter for probiotic lysates, allowing comparable lysate input across experimental groups. Although the selected *Lactobacillus* and *Bifidobacterium* strains were used as commonly used probiotic species, potential strain-specific differences should also be considered in future studies. Furthermore, detailed compositional characterization of probiotic lysates and systematic assessment of batch-to-batch reproducibility were not included in this exploratory study and may warrant further investigation. Additional studies using broader concentration ranges of caffeine and bile acids, together with in vitro release experiments and more physiologically relevant skin models, may further provide could provide a more comprehensive understanding of the effects of probiotic lysates and bile acids on caffeine permeation behavior.

## 5. Conclusions

The results of this study indicate that probiotic bacterial lysates significantly influence the permeation of caffeine across the Skin-PAMPA membrane primarily by altering the permeation profile over time rather than simply reducing overall permeability. Lysate-containing systems showed a lower initial permeation rate followed by a more sustained permeation profile, suggesting that interactions within the donor phase may influence the freely available fraction of caffeine and consequently affect permeation kinetics. In contrast, submicellar concentrations of bile salts increased caffeine permeability, most likely due to their ability to modify membrane properties and enhance molecular diffusivity, with DCA producing a stronger effect than CA. When lysates and bile acids were combined, intermediate permeation profiles were observed, indicating the coexistence of competing mechanisms affecting drug availability and membrane transport.

Although the Skin-PAMPA model represents a simplified system that does not fully reproduce the complexity of human skin, the present findings provide preliminary insight into how biologically derived components may modulate the permeation behavior of small molecules. Overall, these findings may provide a basis for future studies exploring the potential role of biologically active excipients and membrane modulators in the development of topical formulations with tailored permeation behavior.

## Figures and Tables

**Table 1:** Results of SkinSensPred in silico analysis.

Substance	Human Sensitizer (Score)	In AD	In AD (Pesticide)	DPRA /PPRA Sensitizer (Score)	KeratinoSens/LuSens Sensitizer (Score)	h-CLAT Sensitizer (Score)
Caffeine	0.835	Y	N	0.835	0.795	0.805

## Data Availability

Data is contained within the article.
